# Protein expression differs between neural progenitor cells from the adult rat brain subventricular zone and olfactory bulb

**DOI:** 10.1186/1471-2202-9-7

**Published:** 2008-01-16

**Authors:** Martin H Maurer, Robert E Feldmann, Heinrich F Bürgers, Wolfgang Kuschinsky

**Affiliations:** 1 Department of Physiology and Pathophysiology, University of Heidelberg, Im Neuenheimer Feld 326, 69120 Heidelberg, Germany

## Abstract

**Background:**

Neural progenitor cells can be isolated from various regions of the adult mammalian brain, including the forebrain structures of the subventricular zone and the olfactory bulb. Currently it is unknown whether functional differences in these progenitor cell populations can already be found on the molecular level. Therefore, we compared protein expression profiles between progenitor cells isolated from the subventricular zone and the olfactory bulb using a proteomic approach based on two-dimensional gel electrophoresis and mass spectrometry. The subventricular zone and the olfactory bulb are connected by the Rostral Migratory Stream (RMS), in which glial fibrillary acidic protein (GFAP)-positive cells guide neuroblasts. Recent literature suggested that these GFAP-positive cells possess neurogenic potential themselves. In the current study, we therefore compared the cultured neurospheres for the fraction of GFAP-positive cells and their morphology of over a prolonged period of time.

**Results:**

We found significant differences in the protein expression patterns between subventricular zone and olfactory bulb neural progenitor cells. Of the differentially expressed protein spots, 105 were exclusively expressed in the subventricular zone, 23 showed a lower expression and 51 a higher expression in the olfactory bulb. The proteomic data showed that more proteins are differentially expressed in olfactory bulb progenitors with regard to proteins involved in differentiation and microenvironmental integration, as compared to the subventricular zone progenitors. Compared to 94% of all progenitors of the subventricular zone expressed GFAP, nearly none in the olfactory bulb cultures expressed GFAP. Both GFAP-positive subpopulations differed also in morphology, with the olfactory bulb cells showing more branching. No differences in growth characteristics such as doubling time, and passage lengths could be found over 26 consecutive passages in the two cultures.

**Conclusion:**

In this study, we describe differences in protein expression of neural progenitor populations isolated from two forebrain regions, the subventricular zone and the olfactory bulb. These subpopulations can be characterized by differential expression of marker proteins. We isolated fractions of progenitor cells with GFAP expression from both regions, but the GFAP-positive cells differed in number and morphology. Whereas in vitro growth characteristics of neural progenitors are preserved in both regions, our proteomic and immunohistochemical data suggest that progenitor cells from the two regions differ in morphology and functionality, but not in their proliferative capacity.

## Background

Neural stem and progenitor cells have been isolated from various regions of the adult mammalian brain, including the forebrain structures subventricular zone and olfactory bulb. In the subventricular zone and the olfactory bulb, neural progenitor cells are exposed to changing microenvironmental surroundings [1,2]. It is not known whether this exposure is the cause for changes in the composition and function of the progenitor cells. Thus we applied proteomic analysis as large-scale, high-throughput technology based on two-dimensional gel electrophoresis and mass spectrometric peptide fingerprinting, which allows to investigate the expression levels of several hundred of proteins simultaneously [reviewed in ref. [3,4]]. To address the question of regional stem/progenitor cell heterogeneities, we compared the cellular proteomes of neural progenitors isolated from both regions.

In a previous study, we found that differentiation results in changes of the proteome of neural stem cells from the hippocampus [5]. With respect to the present study, not only differentiation, but also migration, local microenvironmental factors or intrinsic genetic programs may influence the proteome of neural progenitor cells.

The subventricular zone and the olfactory bulb are connected by the rostral migratory stream (RMS), a circumscribed pathway allowing neuronal precursors to migrate tangentially from the subventricular zone to the olfactory bulb in a typical chain-like manner [6-8]. Migrating neural progenitors are guided by tubular formations of astrocytes from their origin, the lateral wall of the ventricles, until they reach the olfactory bulb, where they differentiate mainly into interneurons (reviewed in [9,10]).

The guiding astrocytes in the RMS, termed "type B cells", are expressing the glial marker glial fibrillary acidic protein (GFAP) [11]. In the current concept of neurogenesis, these cells contribute to the neural progenitor/neuroblast pool, whether directly, or via a transiently amplifying intermediate form [10]. Doetsch et al. [12] have shown that GFAP-positive cells of the subventricular zone can give rise to neuronal precursors and finally to granular neurons, but the overall contribution of glial cells to adult neurogenesis is still an open question under discussion [10,13,14].

The differentiation pathway from GFAP-positive cells may be only one of several ways by which new neurons can be generated. An alternative pathway of neurogenesis starts from non-GFAP-positive precursors. However, most neurons in the brain seem to be derived from radial glial cells [reviewed in ref. [15]]. It is therefore of interest to know the fraction of GFAP-positive cells in undifferentiated neuronal precursor cells and whether this fraction differs between the subventricular zone to the olfactory bulb.

In the present study, we addressed the question whether neurospheres from the subventricular zone differ in their protein expression pattern from neurospheres from the olfactory bulb. Therefore, we compared the proteomic profiles of neural progenitor cells isolated from the subventricular zone to those of the olfactory bulb of the adult rat brain. Additionally, we compared neurosphere cultures from both regions with regard to the fraction of GFAP-positive cells and their morphology to obtain insight in possible functional differences.

## Results

### Proteomic differences between subventricular zone and olfactory bulb neural progenitors

**Figure 1:**
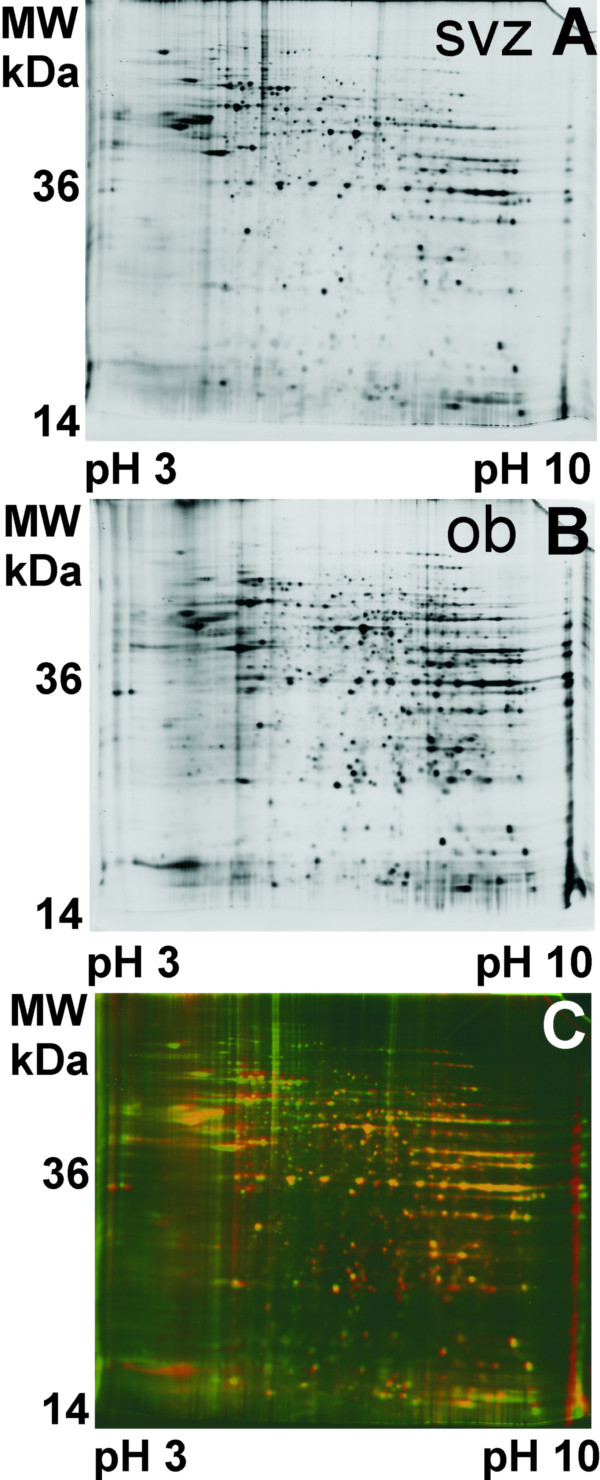
**Original gel images of two-dimensional electropherograms from cultured neurospheres**. (A) Two dimensional gel image of subventricular zone, (B) olfactory bulb neurospheres. The gel images show a similar spot distribution pattern, but distinct heterogeneities in the protein expression profile are obvious. (C) Overlaid gel images, false-color coded, to visualize protein expression differences. Green spots correspond to subventricular zone, red spots to olfactory bulb proteins, yellow spots result from merged spots.

Comparing the progenitor cell proteome of the subventricular zone to that of the olfactory bulb in cultured neurospheres from the adult rat brain, we found major differences in the protein composition, both with respect to protein quantity and quality. We detected a mean ± SD of 968 ± 168 protein spots in gels from the subventricular zone (N = 5 biological replicate gels) and 790 ± 113 in gels from the olfactory bulb (N = 5 biological replicate gels) (Fig. 1). Of these, we did not consider 529 spots for further evaluation, since they were only present in less than 50% of the gels, with 261 spots remaining. Applying Student's t-test for independent groups, we found 74 differentially expressed spots (P < = 0.05), of which 23 protein spots showed lower protein concentrations (down-regulation), whereas 51 protein spots showed higher concentrations (up-regulation) in the olfactory bulb group compared to the subventricular zone group. We identified 15 (65%) of the down-regulated, and 22 (43%) of the up-regulated proteins (Table 1) by mass spectrometry. A total of 105 spots were present solely in the subventricular zone, of which we could identify 18 protein spots (17%) (Table 1).

**Table 1:** Differential expression of proteins in neural progenitors from the subventricular zone and olfactory bulb. Expression factors are normalized by setting the expression of the corresponding protein from the subventricular zone to 1.0. Only proteins with P < 0.05 are displayed.

***Spot ID***	***GenBank Annotation***	***SwissProt accession number***	***Expression factor (olfactory bulb/subventricular zone)***
225	eukaryotic translation elongation factor 1 alpha 2	P27706	3.85
388	pyrroline-5-carboxylate reductase	Q922W5	3.45
267	phosphoglycerate kinase 1	P09411	2.94
314	acetyl-Coenzyme A acetyltransferase 2	Q9BWD1	2.56
21	eukaryotic translation elongation factor 2	P05197	2.33
230	elongation factor 2	P05197	2.17
502	nucleoside diphosphate kinase B	P19804	2.17
185	ATP synthase alpha chain, mitochondrial precursor	P15999	1.85
161	chaperonin subunit 2 (beta)	P80314	1.82
113	chaperonin subunit 5 (epsilon)	P80316	1.75
223	L-arginine:glycine amidinotransferase	P50442	1.75
95	T-complex 1	Q96SF2	1.75
221	elongation factor 1-alpha 1	P20001	1.64
132	protein disulfide isomerase A3 precursor	P11598	1.61
152	3-phosphoglycerate dehydrogenase	O08651	1.59
90	outer dense fiber protein	P97575	1.59
323	isocitrate dehydrogenase 3 (NAD+) alpha	Q9D1L1	1.54
219	L-arginine:glycine amidinotransferase	P50442	1.54
158	3-phosphoglycerate dehydrogenase	O08651	1.52
346	aldehyde reductase 1 (low Km aldose reductase)	P07943	1.52
424	endoplasmic reticulum protein 29	P52555	1.33
339	glyceraldehyde-3-phosphate dehydrogenase	Q9QWU4	1.23
333	cdc42-binding protein kinase beta	Q9Y5S2	0.84
205	enolase 1, alpha	P04764	0.84
300	aldolase C, fructose-biphosphate	P09117	0.80
67	albumin	P02769	0.78
273	acyl Coenzyme A dehydrogenase	P15650	0.69
306	aldolase C, fructose-biphosphate	P09117	0.66
245	creatine kinase	P07335	0.66
60	albumin	P02769	0.63
22	eukaryotic translation elongation factor 2	P05197	0.62
422	phosphoglycerate mutase 1	P25113	0.61
171	Sep-2	Q9ESF7	0.60
618	F1-ATPase chain A	P15999	0.52
470	phosphatidylethanolamine-binding protein	P31044	0.52
96	dihydropyrimidinase related protein-2	P47942	0.51
675	estrogen sulfotransferase	Q9QWS0	0.46
6005	enolase 2	P07323	AS
6004	enolase 2	P07323	AS
5006	glial fibrillary acidic protein	Q9R1Q3	AS
5007	hemoglobin alpha chain	P01946	AS
5003	protein disulfide isomerase A3	P30101	AS
6002	transketolase	P50137	AS
6001	transketolase	P50137	AS
278	acetyl-Coenzyme A acyltransferase 2	P13437	S
279	acetyl-Coenzyme A acyltransferase 2	P13437	S
295	aldolase C, fructose-biphosphate	P09117	S
294	aldolase C, fructose-biphosphate	P09117	S
671	annexin V	P14668	S
194	ATP synthase alpha chain, mitochondrial precursor	P15999	S
6	exocyst complex component Sec15A	O54923	S
187	F1-ATPase, chain A	P15999	S
207	G2/mitotic-specific cyclin B1	P24860	S
351	glyceraldehyde-3-phosphate dehydrogenase	Q9QWU4	S
353	glyceraldehyde-3-phosphate dehydrogenase	Q9QWU4	S
119	HSP60 protein	P19226	S
276	phosphoglycerate kinase 1	P09411	S
275	phosphoglycerate kinase 1	P09411	S
270	phosphoglycerate kinase 1	P09411	S
532	rap7a	Q9QX67	S
786	SH3-domain kinase binding protein 1	Q925R2	S
394	voltage-dependent anion-selective channel protein 1	Q60932	S

S = expression only in the subventricular zone neurospheres. AS = proteins identified additionally in subventricular zone precursors, but no differential expression.

**Figure 2:**
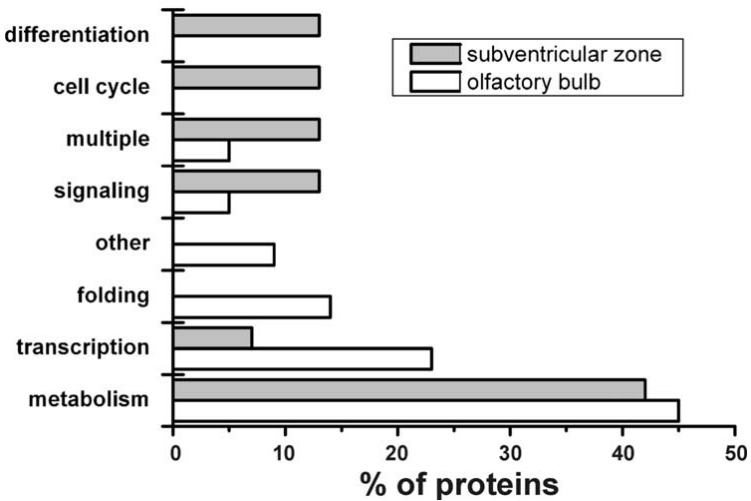
**Functional categories of the identified proteins**. The relative composition of the functional categories varies between the subventricular zone group and the olfactory bulb group. This shift in protein categories may be associated with increased differentiation and integration towards the olfactory bulb. Proteins which have a function in differentiation are highly expressed in undifferentiated cells where differentiation is already initiated. Their expression decreases in terminally differentiated cells, since cellular differentiation programs are not active any more. Therefore, we interpret the decrease in differentiation-related proteins as sign for differentiated, not differentiating cells.

With regard to the two-dimensional gels, the protein expression pattern of neurospheres from the subventricular zone differs significantly from that of neurospheres from the olfactory bulb (Fig. 2). In order to obtain more specific information on functional aspects, we assigned proteins to several functional categories, mainly cell cycle, differentiation, folding, metabolism, signaling, and transcription. The protein expression pattern from the olfactory bulb cells showed more differentially expressed proteins involved in differentiation and microenvironmental integration.

### No differences in population growth, but in marker protein expression

To identify intrinsic differences between the two progenitor cell populations, we compared the growth characteristics in 26 consecutive passages of the neurospheres in both the subventricular zone and olfactory bulb cultures. We did not find differences in the average time for each passage, the number of cells at start and end of the passage, population doublings, population doubling times, and frequency of population doublings (Table 2).

**Table 2:** Growth characteristics of neural progenitors from the subventricular zone and olfactory bulb. Growth characteristics are compared between 26 consecutive passages of neural progenitor cells isolated from the subventricular zone and the olfactory bulb. No significant differences can be seen between the two populations.

***Parameter***	***Subventricular zone***	***Olfactory bulb***	***P-Value***
Number of passages analyzed	26	26	
Average time for passage (d)	16.8 ± 5.0	16.5 ± 5.1	0.843
Number of cells at start of passage (thousands)	250.5 ± 354.9	271.8 ± 355.8	0.830
Number of cells at end of passage (thousands)	11,325.2 ± 8,776.0	13,242.3 ± 10,666.3	0.484
Population doublings	5.5 ± 1.4	5.5 ± 1.4	0.909
Frequency of population doublings	0.4 ± 0.1	0.4 ± 0.2	0.820
Doubling time (d)	2.8 ± 9.5	2.7 ± 6.5	0.483

**Figure 3:**
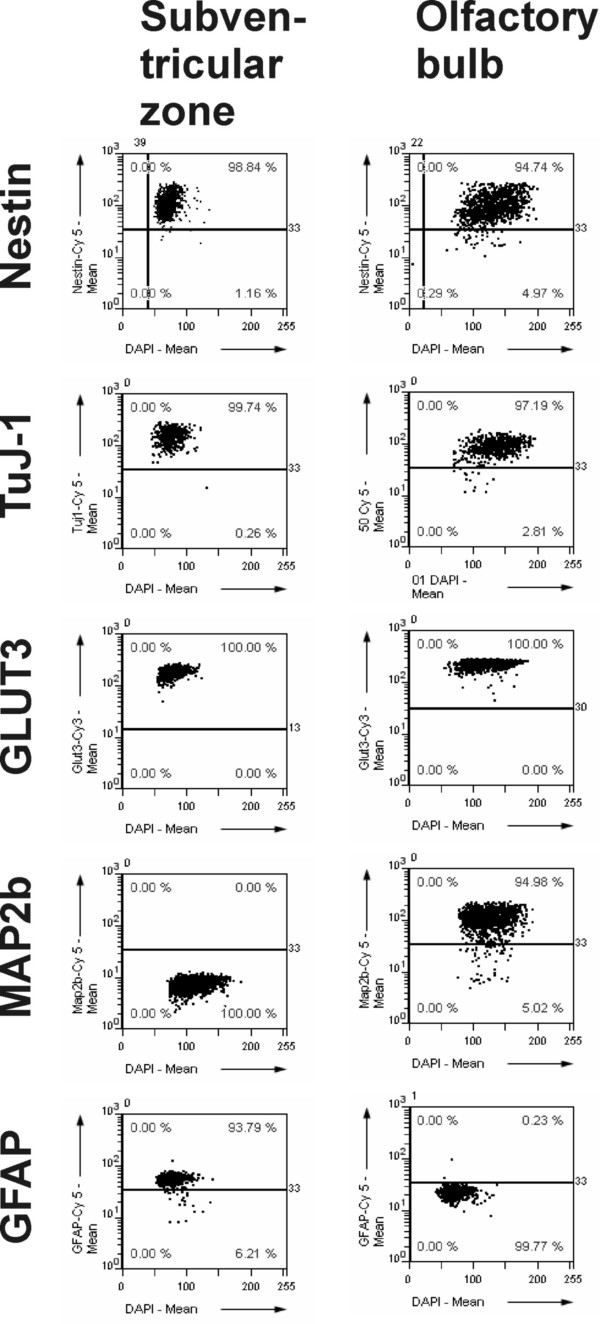
**Molecular marker expression by neural progenitors from the olfactory bulb and subventricular zone**. Comparing the expression of cellular marker proteins for the two neural progenitor populations, we found no significant differences for Nestin, Tubulin-beta-III (clone TuJ-1), and Glucose Transporter GLUT3, whereas the two populations differed in their expression for MAP2b and GFAP.

Additionally, we characterized the two progenitor cell populations by their expression of immunocytochemical marker proteins. Whereas we did not find differences for the expression of the neuronal progenitor cell marker Nestin, and the mature neuronal markers Tubulin-beta-III (clone TuJ-1), and Glucose Transporter GLUT3, we saw differences in the early neuronal marker MAP2b and the astrocyte marker GFAP (Fig. 3). MAP2b expression was not detectable in the subventricular zone progenitors, but provided a strong signal in the olfactory bulb cells.

### GFAP-staining shows qualitative and quantitative differences

GFAP expression was present in the majority of cells of the subventricular zone, but was only minimal in the olfactory bulb progenitors. Using an automated slide and image analysis system for the expression analysis of GFAP in the two populations, we found that about 94% of all progenitor cells from the subventricular zone expressed the astrocytic marker protein GFAP, whereas less than 1% of cells in the olfactory bulb cultures expressed GFAP (P < 0.05).

In the two-dimensional gels, the normalized GFAP spot volume, which was 1.08 ± 1.01 relative units (N = 5 biological replicate gels) in the subventricular zone neurospheres, did not differ from the volume in the olfactory bulb group, which was 0.57 ± 0.29 relative units (N = 5 biological replicate gels) (P < 0.1343).

**Figure 4:**
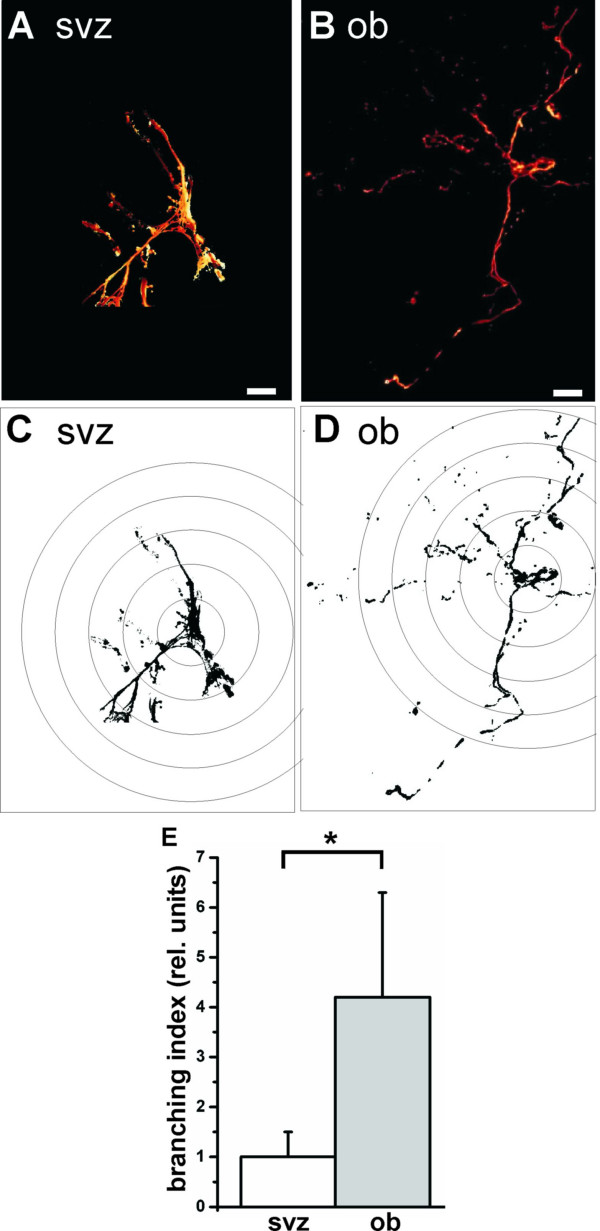
**Morphological dissimilarities between GFAP-positive cells from the subventricular zone and olfactory bulb**. GFAP-positive cells (A) from subventricular zone and (B) from olfactory bulb neurospheres. Cellular morphology and staining result resemble astrocytic fate of the neurospheres. Both cultures showed no difference in the percentage of GFAP-positive cells, but differ in their cellular morphology. (C, D) Binarized images used for branching analysis. The branching index was calculated according to [31], counting dendritic contacts to concentric circles around the cell body with additional weighting for more distant contacts. (E) The GFAP-positive cells in the olfactory bulb show increased branching than the subventricular zone GFAP-positive cells. These results support the impression of a more differentiated state of the olfactory bulb cells (*, P < 0.05; N = 50; Scale bar, 10 μm).

Additionally to the quantitative differences in GFAP expression, we also found morphological differences between the two populations with respect to their site of origin. The GFAP-positive cells in the olfactory bulb showed an increased branching index of 4.2 ± 2.1 compared to the subventricular zone GFAP-positive cells with 1.0 ± 0.5 (P < 0.05; Fig. 4).

## Discussion

### Proteomics

In the present study, we demonstrate major differences between the protein composition of neural progenitor cells dissected from subventricular zone and olfactory bulb. Several factors may contribute to these proteome differences: (1) distinct progenitor cell populations may exist in the subventricular zone and olfactory bulb; (2) the progenitor cell proteome may respond to microenvironmental cues; (3) cytokinesis may change the protein composition of neural progenitor cells on their way along the RMS. We cannot exclude that that there is a spatiotemporal overlap or superposition of the three mechanisms, resulting in a dynamic alteration of the proteomes. Therefore, the changes in the proteome may be explained by all three hypotheses.

#### Growth characteristics

In the current study, we did not detect differences in population growth characteristics between subventricular zone and olfactory bulb neurospheres over a series of 26 passages, but we found differences in the expression of marker proteins. In case of increased differentiation in the olfactory bulb, lower proliferation kinetics could be expected. On the other hand, apoptosis is a second major factor influencing the number of viable cells in both cultures. Thus the unchanged proliferation kinetics may reflect a balanced ratio between proliferation and apoptosis in the neurospheres. Interestingly, neural stem/progenitor cells exhibit a long-term maintenance of their differentiation potential. For example, E14 progenitor cells from the rat striatum did not show differences in gene expression profiles or differentiation potential after 6 years in culture, compared to the original cells [16].

#### GFAP-positive subpopulations

**Figure 5:**
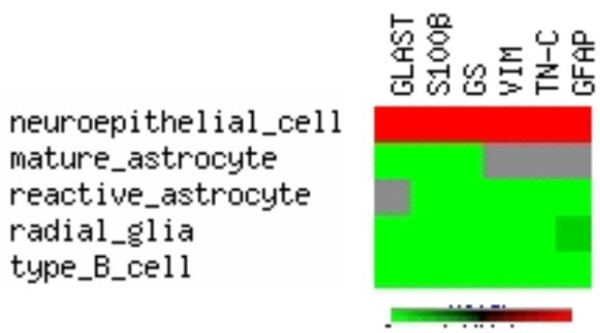
**Expression profiles of glial marker proteins in glial cell populations**. Pseudo-clustering of data from [9, 36]. Red = not expressed, lightgreen = expressed, darkgreen = expessed in some species, grey = no data available or ambiguous data. GLAST = L-glutamate/L-aspartate transporter, GS = glutamine synthase, VIM = vimentin, TN-C = tenascin-C, GFAP = glial fibrillary acidic protein.

In the present study, we found that GFAP was expressed both in the neurospheres from the subventricular zone and the olfactory bulb. Whereas the majority of cells in the subventricular zone expressed GFAP, only a small fraction of cells expressed GFAP in the olfactory bulb cultures. Vice versa, the expression of the neuronal marker MAP2b was stronger in the olfactory bulb cultures. GFAP is generally considered as astrocytic marker [for review, see ref. [3,4,13,17]] (see also Fig. 5). Doetsch et al. [12] reported that GFAP-positive cells in the subventricular zone can give rise to neurons after migrating to the olfactory bulb.

In the present study, we found differences in the quantity and quality of GFAP-positive cells in the olfactory bulb compared to the subventricular zone. There could be two explanations for these findings: First, the GFAP-positive cells in the olfactory bulb retain their immature potential and can give rise to brain cells. In this case, they still have to be considered stem/progenitor cells. Second, cells expressing GFAP in the olfactory bulb are astrocytes. Then GFAP is not only a marker for "precursors" but also a marker of the astrocytic fate in the progenitor cells. Again, we cannot exclude a certain overlap between both explanations, although the GFAP-positive cells showed an astrocytic phenotype which may be explained by spontaneous differentiation in vitro. This is supported by the morphological differences between the GFAP-positive cells from the olfactory bulb compared to those from the subventricular zone (Fig. 4), supporting the impression of a more differentiated state of the olfactory bulb cells, also with regard to the increase in MAP2b-positive cells in the olfactory bulb cells.

In other studies, a regional specificity, but not commitment of neural precursor cells was reported [18,19]. In these studies, the authors proposed that progenitor cells in the subventricular zone are composed of a mixture of cell types, which become "sorted" on their way entering the different migratory pathways, e. g. tangential migration towards the olfactory bulb, or radial migration towards the cortex. In the same context, Fukuda et al. [20] identified neural stem cells in the hippocampus by expression of the marker protein nestin. In their study, some of the nestin-positive cells expressed GFAP additionally, whereas others did not. Both subtypes did also differ in their electrophysiological properties.

Recently, the hypothesis of regionally different neural progenitor populations was further supported by the study of Merkle et al. [21], who provided evidence of distinct progenitor populations already present in the subventricular zone. The data of the present study also imply that not all progenitor cell populations are endowed with similar differentiation potential and that this may result in altered functionality of the cells.

## Conclusion

In the present study, we identified differences on the translational level of neural progenitor cells from two regions of the adult rat brain. Global protein expression profiles of subventricular zone and olfactory bulb precursors differ not only in the quantity of single proteins, but also in their quality: Different protein species of some proteins are expressed between the two groups and some protein species are only expressed in on of the two groups. These data indicate that microenvironmental stimuli, such as growth factors, neurotransmitters, and cell surface molecules influence the proteome in a spatial and temporally restricted manner. Major cellular events such as differentiation, interaction with the extracellular matrix, or cytokinesis may contribute to these changes, since mainly proteins of the cytoskeletal system including motor filaments are differentially expressed. To this end, the progenitor cells from the subventricular zone have to synthesize new cell surface molecules to communicate with neighboring cells and with the guiding astrocytes. In addition, they have to establish new receptors and intracellular signaling pathways to allow them to interact with the cells in their target area. These changes are mirrored by the changes in protein expression.

In the present study, we also report no differences in proliferation and growth characteristics between the subventricular zone and the olfactory bulb, indicating that the proliferative cells of both cultures retained their stem/progenitor potential. On the other hand, we found differences in GFAP expression between the two populations. There are distinct morphological differences between the neurosphere cultures from olfactory bulb and subventricular zone, supporting the idea of at least two distinct progenitor cell populations in the brain, differing in their protein composition.

In conclusion, our data support the hypothesis of several progenitor cell subpopulations endowed with different sets of cellular proteins. It remains unknown whether environmental factors or inherent genetic programs are responsible for the proteome changes described in the present study.

## Methods

### Cell culture

Neural progenitor cells were isolated from the olfactory bulb and the subventricular zone of 4–6 weeks old male Wistar rats as described [5,22]. Protocols are concordant with the policy on the use of animals, as endorsed by the National Institutes of Health, and fulfill the requirements of German law. Briefly, brains washed in 50 mL ice-cold Dulbecco's Phosphate Buffered Saline (DPBS) supplemented with 4.5 g/L glucose (DPBS/Glc). Olfactory bulb and subventricular zone from 6 animals were dissected, washed in 10 mL DPBS/Glc and centrifuged for 5 min at 1600 × g at 4°C. After removal of the supernatant, the tissue was homogenized with scissors and scalpels, resuspended in 20 mL DPBS/Glc, and centrifuged for 5 min at 1600 × g at 4°C. The pellet was resuspended in 10 mL of 0.01% (w/v) papain, 0.1% (w/v) dispase II (neutral protease), 0.01% (w/v) DNase I, 12.4 mM MgSO4 in Hank's Balanced Salt Solution (HBSS), triturated well by a plastic pipette tip, and incubated at room temperature for 40 min. In three washing steps, the homogenate was centrifuged for 5 min at 1600 × g a 4°C and the pellet was resuspended in 10 mL Dulbecco's Modified Eagle's Medium (DMEM)-Ham's F12 medium supplemented with 100 units/mL penicillin, 100 units/mL streptomycin, and 2 mM L-glutamine. Cells were resuspended in 1 mL neurobasal-B27 medium and the cell number was counted. Cells were plated in 2 mL dishes at 200,000 cells in B27-neurobasal medium supplemented with 100 units/mL penicillin, 100 units/mL streptomycin, 20 ng/mL EGF, 20 ng/mL FGF-2, and 2 μg/mL heparin. 4/5 of the medium was replaced weekly, and cells were passaged every 2–2.5 weeks. The neural progenitor cells were maintained for 6–10 weeks in 5% CO2 at 37°C. Population growth parameters were counted as specified in [23].

### Two-dimensional gel electrophoresis

Cells were harvested by centrifugation and proteins were extracted for 60 min at room temperature in a lysis buffer containing 7 M urea, 2 M thiourea, 4% (w/v) CHAPS, 0.5% (v/v) Triton X-100, 100 mM DTT, 0.05% IPG buffer pH 3–10 (GE Healthcare, Amersham Biosciences, Uppsala, Sweden), and 0.156% (w/v) Complete protease inhibitor tablets (Roche, Mannheim, Germany). Sample protein amounts were determined by the Bradford method [24]. Two-dimensional gel elctrophoresis was performed according to [5,22,25]. Briefly, 5–10 μL of the protein solution were suspended in rehydration solution consisting of 6 M urea, 2 M thiourea, 2% (w/v) CHAPS, 0.5% (v/v) IPG buffer pH 3–10, and a few grains of bromophenole blue in a final volume of 350 μL. The samples were applied to 18 cm pH 3–10 non-linear gradient IEF gel strips for isoelectric focussing the IPGphor apparatus (GE Healthcare, Amersham Biosciences, Uppsala, Sweden). The IEF gel strips were allowed to reswell for 12 hours at 30 V. Then 200 V, 500 V and 1000 V were applied for 1 hour each. Voltage was increased to 8000 V in 30 min and kept constant at 8000 V for 12 hours, resulting in a total of 100,300 Vh. Gel strips were equilibrated for 20 min each in an SDS equilibration buffer consisting of 50 mM Tris-HCl, pH 8.8, 6 M urea, 30% (v/v) glycerol, 2% (w/v) SDS, a few grains of bromophenole blue, and 1% (w/v) dithiothreitol or 2.5% (w/v) iodoacetamide, respectively. The second dimension separation was performed using 12.5% polyacrylamide gels in the presence of 0.1% (w/v) sodium dodecylsulfate. The gels were run at 30 mA for 30 min and 100 mA for about 4 h in a 20 cm × 20 cm water-cooled vertical electrophoresis apparatus (OWL, Woburn, MA, USA). For image analysis, gels were silver stained [26]. Briefly, gels were fixed over-night in 40% (v/v) methanol containing 10% (v/v) acetone. After 3 times of washing in 30% (v/v) ethanol for 20 min each, gels were impregnated in 0.02% (w/v) sodium thiosulfate. Then the gels were washed quickly in bidestilled water and stained with 0.2% (w/v) silver nitrate for 20 min. Again the gels were washed quickly in bidestilled water and carefully developed in 3% (w/v) sodium carbonate, 0.05% (v/v) formole 37%, 0.0005% (w/v) sodium thiosulfate. After washing 3 times with bidestilled water, the reaction was stopped by 0.5% (w/v) glycine.

### Two-dimensional gel image analysis and mass spectrometry

Gels were scanned and images were analyzed by the Phoretix 2D Advanced software (Nonlinear Dynamics, Newcastle-upon-Tyne, UK). Statistical analysis of the spot volumes defined as integral of spot area multiplied by optical density was done comparing normalized means ± standard deviations from 5 gels of each group by Student's t-test for unpaired data [27]. Three-dimensional reconstruction of protein spot volumes was done as reported [5]. Spots of interest were excised and digested by trypsin for mass spectrometry. Peptide mass fingerprinting (PMF) using MALDI-TOF, peptide sequencing using LIFT-TOF/TOF, data acquisition, and database search was performed by the mass spectrometry group at ProteoSys (Mainz, Germany) using an Ultraflex TOF/TOF (Bruker-Daltonics, Bremen, Germany) as described priviously [27]. Mass spectra were identified by searching the NCBI non-redundant protein database using Mascot [28,29].

### Immunohistochemistry

Several drops of cell culture medium containing neurospheres and single cells either from subventricular zone or olfactory bulb were placed on a glass slide, sealed with a cover slip and frozen at -80°C. For staining, the cover slip was removed quickly and the cells on the slide were fixed for 10 min in 4% paraformaldehyde in PBS, pH 7.4, with 0.2% Tween-20 (PBST). Slides were blocked 1 h at room temperature in 5% milk powder in PBST and incubated with antibodies against the neural progenitor marker nestin (1:100, BD Biosciences, Heidelberg, Germany), the astrocytic marker GFAP (1:100; BD Biosciences, Heidelberg, Germany), the neuronal markers tubulin-beta-III (1:500; clone TuJ-1; Abcam, Cambridge, UK), microtubulus-associated protein MAP2b (1:100; Chemicon, Cambridge, UK), and Glucose Transporter GLUT3 (1:250; Biogenesis, Newcastle-upon-Tyne, UK) overnight at 4°C in a humidified chamber. The next day, slides were washed 3 times for 5 min in PBST and incubated with a suitable Cy3- or Cy5-conjugated secondary antibody (1:200, Jackson ImmunoResearch, West Grove, PA, USA) for 3 h at room temperature. After washing 3 times for 5 min each with PBST, nuclei were stained with 4',6-diamidino-2-phenylindole (DAPI, 1:5,000, Molecular Probes, Eugene, OR, USA) for 5 min. Slides were embedded in fluoprotective mounting media [30] and stored at 4°C in the dark until fluorescence microscopy was performed.

### Image analysis

#### Cytometry and cell differentiation

Images were recorded using a digital camera (DC500, Leica, Bensheim, Germany) on a fluorescent microscope (DM-R HC, Leica Microsystems, Bensheim, Germany; BX50, Olympus, Hamburg, Germany). For image cytometry, images were analyzed using the TissueQuest software (TissueGnostics, Vienna, Austria). Briefly, nuclei were detected by dissection algorithms in the DAPI channel. Then, immunopositive cells were detected by a non-annular signal grow algorithm around the nuclei. The signals for the respective primary antibody were plotted against the nuclear DAPI signals to create FACS-like scattergrams [25].

#### GFAP subpopulations

Using the grain counting option of the MCID Elite software, version 7.0 (GE Healthcare, ImagingResearch, St. Cathrines, Ontario, Canada), and a digital camera (CoolSnap cf, Roper Scientific, Tucson, AZ, USA), we took automated tiled field images on a fluorescent microscope (Leica Microsystems, Bensheim, Germany). Grain counting was performed to get target information for GFAP and DAPI signals. The ratio of (GFAP-positive cells):(number of nuclei counted on the slide) was compared between subventricular zone and olfactory bulb neural progenitor cells in at least 6 slides. Confocal images were obtained by the Leica Confocal Microscope TCS SP II (Leica Microsystems, Bensheim, Germany). Branching indexes were counted using the algorithm from [31-33]. Briefly, circular fields are drawn around the cell nucleus. Each dendrite crossing the boundary is scored and weighted for distance. Values were normalized to the subventricular zone cells. Fifty cells each from olfactory bulb and subventricular zone were counted.

### Statistical Analysis

Results of two groups were compared by using Student's t-test, whereas multiple groups were compared using ANOVA (SPSS version 9, Chicago, IL). The level of significance was set to P < 0.05. Proteomic data were evaluated as described elsewhere in detail [27,34,35].

## List of abbreviations

GFAP: Glial fibrillary acidic protein;

GLUT3: Glucose transporter molecule 3;

MALDI-TOF: Matrix-assissted laser desorption/ionnization time-of-flight mass spectrometry;

MAP2b: Microtubulus-associated protein 2b;

RMS: Rostral migratory stream;

## Authors' contributions

MHM and REF carried out the proteomic experiments, MHM and HFB did the immunocytochemical analysis. MHM analyzed the growth conditions, performed the statistical analysis and wrote the manuscript. WK participated in the design of the study, conceived of the study, and participated in its design and coordination and drafted the manuscript. All authors read and approved the final manuscript.
